# Clinical manifestations and cross-reactions between cockroaches and termites and identification of termites’ major allergens

**DOI:** 10.1371/journal.pone.0342319

**Published:** 2026-03-23

**Authors:** Mey-Fann Lee, Chi-Shen Wu, Tzu-Mei Lin, Hou-Feng Li, Yi-Hsing Chen

**Affiliations:** 1 Department of Medical Research, Taichung Veterans General Hospital, Taichung, Taiwan; 2 Division of Allergy, Immunology and Rheumatology, Taichung Veterans General Hospital, Taichung, Taiwan; 3 Department of Entomology, National Chung Hsing University, Taichung, Taiwan; 4 i-Center for Advanced Science and Technology (i CAST), National Chung Hsing University, Taichung, Taiwan; 5 School of Medicine, National Chung Hsing University, Taichung, Taiwan; 6 Center of Rheumatology and Immunology, China Medical University Hospital, Taichung, Taiwan; Purdue University, UNITED STATES OF AMERICA

## Abstract

The American cockroach (*Periplaneta Americana,* CRa A) is a strong risk factor for allergic sensitization and asthma morbidity. Molecular phylogenetic studies demonstrated termites are social cockroaches specialized in feeding on plant materials. We hypothesized that termite allergies may be misdiagnosed as cockroach allergies due to cross-reactivity in routine clinical tests. Herein, we identified the allergenic cross-reaction between CRa A and termites and determined the clinical significance of termite allergies. Sixteen subjects with a history of exposure to termites, nine (56%) had a positive immediate skin reaction to *Coptotermes formosanus* or *Coptotermes gestroi*, but only two individuals were reactive to CRa A. Among the termite-allergic subjects, 8 had rhinitis and one had dermatitis, which manifested as itchy eczematous skin rashes. Immunoblotting revealed 7 allergenic components could bind specific IgE antibodies in 8 termite-allergic patients. The major allergens of 30/32-, 43-, and 70-kDa were Copt f 7, Copt f 9, and Copt f 3 by immunoblot inhibitions. Antibodies against Per a 3, Per a 4, and Per a 6 recognized bands in both extracts of cockroach and alate termites, indicating a more similar gene expression between alate termites and cockroaches compared to worker and soldier castes. Our study underscores the importance of recognizing termites as a novel indoor allergenic source and the necessity for further research to develop effective diagnostic and therapeutic strategies.

## Introduction

The past decade has seen a concerning rise in allergic diseases, posing significant global public health challenges [1,2]. Indoor allergens gained particular importance during the COVID pandemics as people spent extended periods indoors, increasing their exposure to indoor inhalant allergens [3,4]. In Taiwan, the American cockroach (*Periplaneta americana*) ranks as the second most prevalent aeroallergen, surpassed only by house dust mites [5–10]. A comprehensive study of allergen sensitization in China revealed the cockroaches were the most common allergen (27.0%) in Southwest China, followed closely by house dust mites (25.6%) [11]. Since allergen avoidance can effectively reduce allergic symptoms, research focused on tracking changes in allergen sensitization patterns is essential for updating personalized treatment approaches [4].

Cockroach allergens in homes represent one of the strongest risk factors for allergic sensitization and asthma severity in children [12,13]. The domestic presence of American cockroaches varies geographicaly. United States surveys detect these allergens in 20–48% of homes without visible signs of infestation [14,15], while Korean studies found cockroaches in 62% of the sample homes [16]. Our research revealed cockroach allergens in 100% of dust samples from Taichung Taiwan households [9]. Notably, cockroach allergens can trigger IgE production and severe asthma at concentrations 10–100 times lower than cat or mite allergens [17]. Recently, the WHO/IUIS Allergen Nomenclature database (www.allergen.org) has expanded to include 20 allergen groups from *P. americana*, with 9 new additions in the past five years [6,7,9,10,18–20].

Termites are recognizable structural pests adapted to urban areas, making them an increasing pest problem worldwide including Taiwan [21]. Recent molecular phylogenetic studies demonstrated that termites comprise a monophyletic group embedded within diverse cockroach taxa [22,23]. In another words, termites are social cockroaches that specialize in feeding on plant materials. Cockroaches are a well-known source of indoor aeroallergens and termites have also recently been considered potential allergenic insects [24,25]. Formosan subterranean termites, *Coptotermes formosanus* Shiraki, and Asian subterranean termites, *Coptotermes gestroi* (Wasmann), are the two most destructive termite pests worldwide. Rust and Su emphasized their rapid spread across the southern United States, the Caribbean, and parts of South America, where they increasingly cause severe structural damage [26]. Recent citizen-science surveys also confirm widespread establishment in Taiwan, and reports document expansion into China, Japan, and other subtropical and tropical regions [21]. In addition, during swarming season, winged termite, alate, are attracted to artificial lights, potentially spreading allergens in urban environments.

We hypothesized that termite allergies may be misdiagnosed as cockroach allergies due to cross-reactivity in current routine clinical tests. In this study, we aimed to define the allergenic cross-reaction between American cockroaches and termites and to determine the clinical significance of termite allergies.

## Materials and methods

### Preparation of whole-body extracts of the three major castes from two species of termites

A *C. formosanus* colony (FF165) was raised from a pair of winged sexual forms, alates, collected in Taichung City, on May 22, 2017 (TW4813). A *C. gestroi* colony (GG068) was raised from a pair of alates collected in Tainan City, on April 19th, 2017 (TW4753). In termite colonies, the major castes are the reproductive (winged alate or dealate), soldier, and worker castes, with workers making up about 90% of a colony. In this study, we examined all three castes for the first time.

For protein extraction, 1 gram of termites from each caste was pulverized in a mortar with 5 ml of phosphate-buffered saline (PBS) containing protease inhibitor cocktail (Biotools, Taiwan). The preparation was extracted overnight at 4°C and then centrifuged at 10,000 g for 30 min to remove insoluble materials. The supernatant was filtered through a 0.45-μm pore filter and frozen at −70°C until further usage. The protein content of the extracts was determined using a protein assay dye reagent purchased from Bio-Rad (Richmond, CA, USA), according to the manufacturer’s instructions.

### Preparation of American cockroach allergens (Cra A)

CRa A was prepared from *Periplaneta americana* by extracting the whole body of insects with Coca’s solution in our laboratory [27].

### Skin prick test

Sixteen subjects with a history of termite exposure from a termite laboratory at National Chung Hsing University (6 females and 10 males, ranging from 21–63 years) were enrolled in this study from 7^th^ September 2022–6^th^ September 2024. The subjects completed an allergy questionnaire and performed skin prick tests after signing written informed consent in the Allergy Clinic of Taichung Veterans General Hospital (TCVGH-IRB No.：CE22339A).

There are four lab-made crude extracts used in the skin prick test (SPT): American cockroach (CRa A) [27], *Forcipomyia taiwana* (For t) [28], and termite extracts (FF165, GG068), prepared as previously described. In this study, a commercially available SPT reagent for *Dermatophagoides pteronyssinus* (D1, Stallergenes-Greer, Lenoir, NC) was also used. Protein samples prepared in the lab at concentrations of 200 µg/mL in PBS containing 50% glycerol were used for SPT with epicutaneous sterile disposable Sharp Test® applicators (Greer Laboratories). Histamine (1 mg/ml) and 50% PBS-glycerol were used as positive and negative controls, respectively. All skin test results were read 20 minutes after placement. A response with a wheal or an erythema 3 mm larger than that produced by the negative control, but less than 1/2 of the diameter of the positive control, was graded as 1 + , and a reaction ranging between 1/2 and equivalent size of the positive control was graded as 2 + . A reaction compatible with the positive control was graded as 3 + . A reaction larger than the positive control was graded as 4 + .

### Specific IgE detection assay

Serum samples were submitted to the automated microfluidic-based immunoassay system (BioIC, lab-on-chips) [29] to detect specific IgE antibodies against 40 individual allergens as follows: 16 inhalant allergens (Dermatophagoides pteronyssinus, Dermatophagoides farinae, Alternaria alternata, vernal grass, cocksfoot, timothy, rye, alder, birch-tree, hazel, oak, mugwort, plantain, rape, cat, dog), 16 food allergens (egg white, cow’s milk, cod, rice, peanut, soya, hazelnut, tomato, carrot, potato, egg yolk, α-lactalbumin, β-lactoglobulin, casein, banana, mix of citrus) and 1 contact allergen (latex) were chosen for comparison. The cut-off for a positive result for BioIC assays is 0.35 kU/l.”

### IgE immunoblotting and inhibition assay

Termite extracts were loaded on a 4% polyacrylamide stacking gel with a 12% separating gel, and the gel was run with a discontinuous buffer by Laemmli’s method. After electrophoresis, gels were fixed and stained with 0.2% Coomassie brilliant blue R250. For immunoblotting, gels were transferred electrophoretically to nitrocellulose membranes (Millipore). After transfer, the membranes were blocked with 5% skimmed milk in PBST (PBS containing 0.05% Tween 20) for 2 h at room temperature. Blots were incubated with individual serum (diluted 1:10) from termite-allergic subjects overnight at 4°C. The blots were washed for three 20-minute periods in PBST and then incubated with 1:4000 dilution of mouse anti-human IgE horseradish peroxidase (HRP) conjugate (Abcam) for 2 h at room temperature. After extensive washing, the membrane was developed using an enhanced chemiluminescent solution (ECL) (Millipore, MA, USA), and the signals were recorded by exposure to a G:BOX Chemi XX9 gel imaging system (Syngene, Cambridge, UK). For immunoblotting inhibition, the human sera were mixed with an equal volume of a specific recombinant allergen (150 μg/ml) at 4**°**C overnight, and immunodetection was then performed as described above.

### Immunodetection of termite extracts with individual Per a 1–10 polyclonal antibodies and immunoblotting inhibition for cross-reactivity between termite and American cockroach allergens

Lab-produced rabbit anti-Per a 1–10 antibodies have demonstrated high specificity for allergens of American cockroaches by immunoblotting without cross-reaction with other cockroach proteins [9]. In this study, those antibodies were used for the immunodetection of termite extracts to evaluate the cross-reaction between termites and cockroaches. After protein transfer, the nitrocellulose membranes (Millipore) were blocked with 2% bovine serum albumin for 1 h and then probed with individual rabbit anti-Per a 1–10 antibodies for another 2 h. Subsequently, the membranes were incubated with peroxidase-conjugated goat anti-rabbit IgG antibody (10,000-fold dilution, Millipore) for 1 h, and then developed using ECL solution (Millipore).

### Cloning and sequencing of termite major allergens Copt f 3, f 7, and f 9

The *C. formosanus* cDNA library was used as a template for PCR-based cloning of the termite major allergens Copt f 3, f 7, and f 9. Sequences of the primers and the theoretical size, pI, and molecular weights of the cloned allergens are listed in **S1 Table**. The PCR products were purified by a BandPrep kit (Genepure, Taichung, Taiwan) and ligated into a pCR2.1 TA vector (Invitrogen, CA, USA). Then, sequences of the 4 cloned cDNAs were determined using an automatic DNA sequencer (Applied Biosystems, CA, USA).

## Results

### Cross-reactive patterns between American cockroaches and three castes of termites

**Fig 1:**
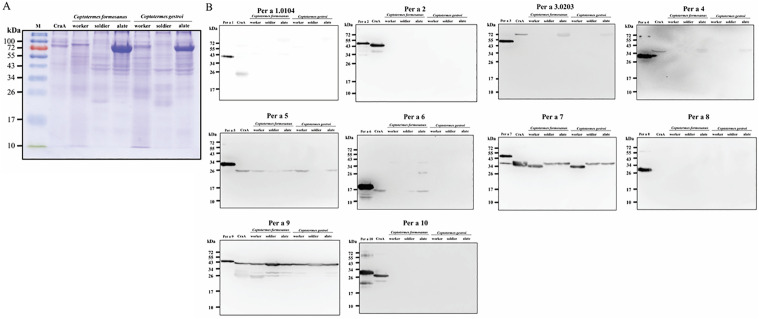
(A) Coomassie blue-stained SDS-PAGE and (B) immunoblotting of crude extracts from cockroaches (CRa A) and major castes of 2 termites with rabbit anti-Per a 1-10 antibodies, respectively. *E.coli*-expressed proteins of Per a 1 to 10 were used as the positive control in each panel, respectively. *f.* represents *C. formosanus; g.* represents *C. gestroi.*

We previously reported a difference in the pathogenicity of different allergen components from American cockroaches [6]. To confirm the comprehensive cross-reaction between termite proteins and cockroach allergens (CRa A), lab-prepared termite extracts of 3 castes from *C. formosanus* and *C. gestroi* were probed with individual antibodies against Per a 1–10 [9], respectively. Immunoblotting data are shown in **Fig 1** and summarized in **Table 1**. Tropomyosin (e.g., Per a 7) and arginine kinase (e.g., Per a 9) are major allergens in cockroaches, house dust mites, and shellfish and are considered pan-allergens. As expected, rabbit anti-Per a 7 and Per a 9 antibodies specifically recognized and bound to an identical band in all 7 extracts from CRa A and 2 termites. Interestingly, the Per a 1, Per a 2, and Per a 10 antibodies each recognized a dominant band only in the CRa A lane, but no signal was detected in the other six extracts from 2 termites. Antibodies against Per a 3, 4, or 6 only recognized a single band on lanes of CRa A and alate termites. This indicates that the gene expression of alate termites showed greater similarity than the other two castes, workers and soldiers when compared with the gene expression of cockroaches. Per a 8 is present at levels too low to be consistently detected in crude extracts. Overall, the immunoblot findings provide evidence that termite proteins may act as allergens, and the species of *C. formosanus* was more cross-reactive with American cockroaches than *C. gestroi*, especially for Per a 5 and Per a 6.

**Table 1:** Cross-reactivity of allergens between American cockroaches and termites by immunodetection.

Allergens	Biochemical name	M.W. (kDa)	Prevalence of Per a 1–10 by our team (%)	*Coptotermes formosanus/ Coptotermes gestroi* (castes of worker, soldier, alate)
Per a 1	Midgut microvilli protein homolog	26-45	54-77	**N/N**
Per a 2	Inactive aspartic protease	36	64	**N/N**
Per a 3	Insect hemolymph	56-79	26-95	Y(alate)/Y(alate)
Per a 4	Lipocalin	21	43	Y(alate)/Y(alate)
Per a 5	Glutathione S-transferase	28.9	62	**Y(All)**/ Y(worker, alate)
Per a 6	Troponin C	18	52	Y(alate)/ N
**Per a 7**	**Tropomyosin**	33	52	**Y (All)/Y (All)**
Per a 8	Myosin light chain	22.6	20	ND^1^
**Per a 9**	**Arginine kinase**	43	55	**Y (All)/Y (All)**
Per a 10	Serine protease	28	49	**N/N**

^1^ND: not determined (due to Per a 8 being too rare to be detected in all crude extracts).

### Demographic and clinical characterization of termite allergic subjects

Of the 16 subjects, 8 and 6 individuals gave a positive immediate skin reaction to *C. formosanus* or *C. gestroi*, respectively. Demographic data and clinical findings are summarized in **Table 2**. Eight of the 9 termite-SPT positive subjects had symptoms of allergic rhinitis, and one had only allergic dermatitis, according to the results of the questionnaire. Two of seven termite-SPT negative subjects had allergic rhinitis due to mite (*D. farinae*) based on a BIO-IC allergen blood test. Unexpectedly, there were only two positive reactions of SPT to CRa A among eight *C. formosanus*-SPT positive subjects, but there were up to seven positive reactions to *D. pteronyssinus* (D1). We speculate that termites may have effects beyond pan-allergenic cross-reactivity, and could play a role in the initial sensitization of the allergic response via repeated exposure.

**Table 2:** Results of skin prick test and blood test for allergen diagnosis by BIO-IC.

Subject	Results of skin prick test (SPT)					Allergen diagnosis by BIO-IC	Symptoms to termites
No. (Age/sex)	D1	For t	CRa A	FF165	GG068	Item(class)	(Allergy questionnaire)
1 (36/M)	4+	3+	4+	4+	3+	D1(3), D201(1), G2(2)	Allergic dermatitis
2 (26/F)	4+	–	–	2+	–	D1(6), D2(6), D201(3), F2(2)	Allergic rhinitis
3 (27/F)	–	–	–	4+	3+	All Negative	Allergic rhinitis
4 (22/M)	3+	–	–	2+	2+	D1(3), D2(3), D201 (2), F23(2), F24(2)	Allergic rhinitis
5 (26/M)	3+	4+	3+	4+	3+	D1(3), D2(3), D201(3), F23(2), F24(2)	Allergic rhinitis
6 (42/M)	3+	–	–	3+	–	D1(2), D2(2)	Allergic rhinitis
7 (23/M)	2+	–	–	2+	3+	D1(2), D2(2)	Allergic rhinitis
8 (24/M)	3+	–	–	3+	–	E1(4)	Allergic rhinitis
9 (22/M)	2+	–	–	–	2+	D1(3), D2(3), E1(2), F20(2), F47(2), G2(2), G6(2), K82(2), T19(2), W1(2), W12(2)	Allergic rhinitis, Urticaria
10 (45/M)	–	–	–	–	–	All Negative	None
11 (22/F)	–	–	–	–	–	All Negative	None
12 (30/F)	–	–	–	–	–	All Negative	Allergic rhinitis
13 (21/M)	–	–	–	–	–	D2(1)	Allergic rhinitis
14 (63/F)	–	–	–	–	–	All Negative	None
15 (57/F)	–	–	–	–	–	All Negative	None
16 (23/M)	3+	–	–	–	–	D2(1)	Allergic rhinitis
Positive rate of SPT	**9/16** **(56%)**	**2/16** **(13%)**	**2/16** **(13%)**	**8/16** **(50%)**	**6/16** **(38%)**	D1: *D. pteronyssinus*, D2: D. farinae, D201: Blomia tropicalis,E1: Cat dander, F2: Milk, bovine, F20: Almond, F23: Crab, F24: Shrimp, F47: Garlic, G2: Bermuda, G6: Timothy, K82: Latex, T19: Acacia, W1: Ragweed, W12: Goldenrod.	

### The IgE-binding pattern of termite proteins from caste workers by immunoblotting

**Fig 2:**
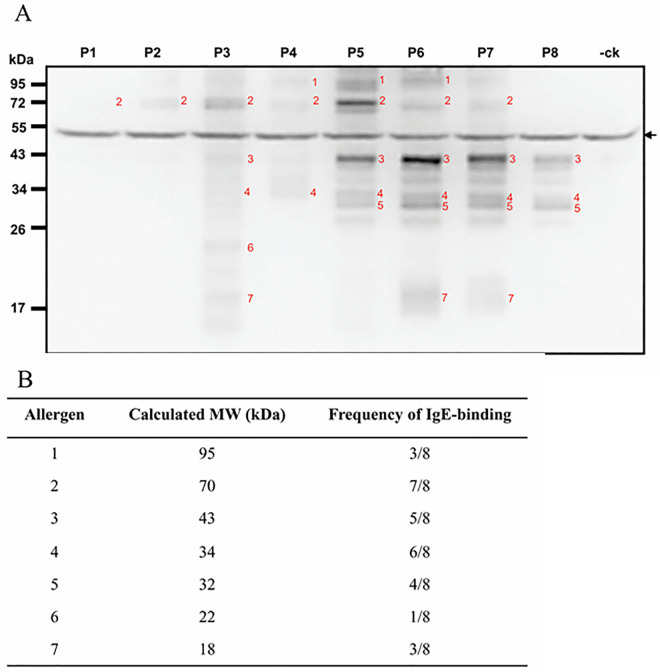
(A) Immunoblot patterns of crude extract from *Coptotermes formosanus* workers with termite-allergic subjects (P1-P8) and a non-atopic subject (-Ck). The numbers at left indicate the size of standard proteins in kDa. The black arrow indicates a non-specific band at the site of 50 kDa. **(B)** List of predicted allergens in sequential molecular weight and the IgE-binding frequency among 8 tested patients.

In a termite society, workers comprise around 90 ~ 98% of individuals in a colony [30]. We chose to study the allergenic profile of termite proteins from workers. Immunoblotting revealed that 7 allergenic components were able to bind specific-IgE antibodies of 8 termite-allergic patients (**Fig 2A**). Allergens of 18, 22, 30, 32, 43, 70, and 95 kDa bound 37.5% (3/8), 12.5% (1/8), 50% (4/8), 62.5% (5/8), 62.5% (5/8), 87.5% (7/8), and 37.5% (3/8) of the allergic sera, respectively. The results are summarized in **Fig 2B**. Notably, there was a nonspecific binding band around 50 kDa found in both termite-allergic and non-allergic sera. Data revealed that the four proteins of 30, 32, 43, and 70 kDa are major allergens of *C. formosanus,* with a prevalence greater than 50% among the tested sera. Furthermore, we demonstrated that the major allergens of 30/32-, 43-, and 70-kDa were Copt f 7, Copt f 9, and Copt f 3 by immunoblot inhibitions using Per a 7, Per a 9, and Per a 3 as inhibitors, respectively. A representative image is presented in S1 Fig.

### Alignments of the deduced amino acid sequences of Copt f 7n, f 9n, and f 3n with known arthropod allergens

**Fig 3:**
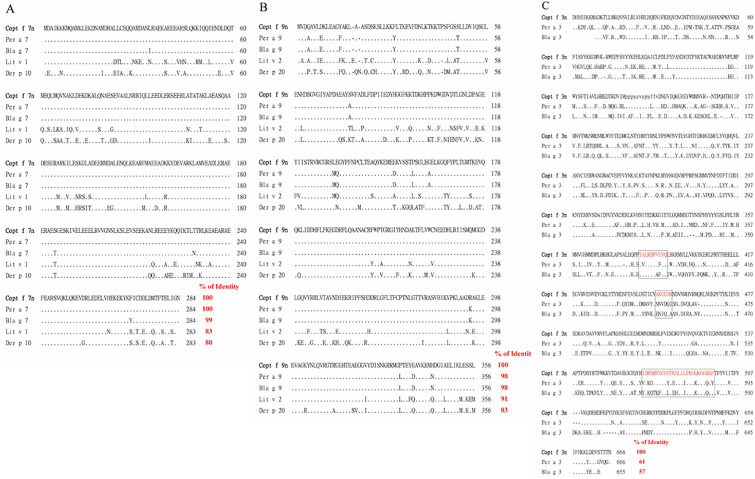
Comparison of (A) Copt f 7n, (B) Copt f 9n, and (C) Copt f 3n with the allergenic sequences from *Periplaneta americana, Blattella germanica, Litopenaeus vannamei, and Dermatophagoides pteronyssinus.*

The results of nucleotide sequencing and computer-assisted homology search revealed that there were only 1, 2, and 6 different nucleotides of novel cloned Copt f 7n (855 bp), Copt f 9n (1071 bp), and Copt f 3n (2007 bp) from the published termite tropomyosin (GenBank no. KC571878.1), arginine kinase, [25] and hemocyanin-like protein (GenBank: KF718963.1), respectively. However, all of the deduced amino acid sequences of Copt f 7n/284 aa, Copt f 9n/356 aa, and Copt f 3n/668 aa matched exactly. Alignments of protein sequences of Copt f 7n, Copt f 9n, and Copt f 3n with the respective arthropod allergens from *Periplaneta americana* (Per a)*, Blattella germanica* (Bla g)*, Litopenaeus vannamei* (Lit v)*, and Dermatophagoides pteronyssinus* (Der p) are presented in **Fig 3**. **Fig 3A** shows termite tropomyosin had high conservation with 99–100% amino acid identity to cockroach Per a 7 and Bla g 7, with 80–83% identity to shrimp Lit v1 and mite Der p 10. **Fig 3B** shows termite arginine kinase homolog with 98% amino acid identity to cockroach Per a 9 and Bla g 9, with 83–91% identity to shrimp Lit v 2 and mite Der p 20. The comparison of the deduced amino acid sequence of termite hemocyanin-like protein with cockroach Per a 3 and Bla g 3 revealed only 61% and 57% identities, respectively (**Fig 3C**).

## Discussion

Accurate identification of specific IgE against the causative allergens and precise knowledge of potential allergic cross-reactivities are required for the optimal clinical management of allergies [31]. There are strong links between cockroach allergy and conditions such as asthma, allergic rhinitis, and atopic dermatitis [6,18,27,32]. Termites are eusocial cockroaches that diverged within the Blattodea. The three allergenic sequences of termites are more similar to those of the American cockroach than to those of the German cockroach, consistent with the phylogenetic placement of termites as being more closely related to the family Blattidae than to the family Ectobiidae [33]. In termite colonies, a hierarchical caste system exists, which consists of three main castes – workers, soldiers, and alates. Our study examined the allergenic cross-reactivity between American cockroaches and termites by investigating the protein extracts from the three major castes of two termite species, *C. formosanus* and *C. gestroi*. Previous studies have indicated the presence of major allergens such as tropomyosin (Per a 7) and arginine kinase (Per a 9) in cockroaches, house dust mites, and shellfish, which are known to be pan-allergens due to their cross-reactivity [24,34]. Our findings support this notion, as rabbit anti-Per a 7 and Per a 9 antibodies specifically recognized identical bands in all termite extracts, confirming the presence of these major allergens. However, we emphasize that *in vitro* IgE binding does not necessarily translate into clinically symptomatic cross-reactivity. Additional investigations are warranted to clarify this relationship.

Termites eat cellulose-based materials, while cockroaches are omnivorous [35]. Cockroaches produce a wide variety of allergens in different parts of the body, which defines how they are released to the environment [20]. Previously, our study found the presence of Per a 1, 2, and 10, predominantly in roach feces [9]. Interestingly, antibodies against Per a 1, Per a 2, and Per a 10 recognized dominant bands only in American cockroach extracts, with no detectable signals in termite extracts. Per a 1 isoallergens are homologous to midgut microvilli proteins. Per a 2 and Per a 10 are proteolytic enzymes predominantly located in the hindgut and feces of cockroaches, which eat almost anything [9,10]. This suggests that certain allergens are unique to cockroaches, while others are shared with termites. Since these three allergens primarily originate from the digestive system, the differences likely arise from the distinct digestion systems and feeding habits of the two insects.

The antibodies against Per a 3, Per a 4, and Per a 6 recognized bands in both cockroach and alate termite extracts, but not the worker and soldier castes. Termites are social insects and despite having multiple castes in a given species, all of those castes share the same genome. The results indicate the gene expressions of hemolymph, lipocalin, and troponin C are different between the reproductive caste, alates, and the two sterile castes, workers and soldiers. Per a 5 is a glutathione S-transferase associated with a detoxifying function and Per a 6 is a part of the muscle components [9,20]. Our data demonstrated that *C. formosanus* showed more significant cross-reactivity with American cockroaches than *C. gestroi*, particularly based on the presence of Per a 5 and Per a 6. This suggests species-specific differences in allergenic potential and cross-reactivity, highlighting the need for species-specific diagnostic tools in allergy testing.

The skin prick test (SPT) results revealed that 8 out of 16 subjects had positive reactions to *C. formosanus* extracts, while 6 subjects reacted positively to *C. gestroi*. Among the termite-allergic subjects, 8 had symptoms of rhinitis and one had dermatitis that manifested as itchy eczematous skin rashes. Interestingly, only two subjects showed positive SPT reactions to American cockroach allergens, while seven reacted to *D. pteronyssinus* (house dust mites), suggesting that termite sensitization might be a distinct allergic response, rather than being solely due to cross-reactivity with common indoor allergens.

These findings underscore the importance of considering termites as a novel indoor allergenic source capable of primary sensitization and further indicate that specific diagnostic methods are needed to accurately identify termite allergies. Given that worker termites constitute approximately 90% of a colony [30], we focused on the allergenic profile of worker caste proteins. Immunoblotting revealed seven allergenic components binding specific IgE from termite-allergic patients, with proteins of 30, 32, 43, and 70 kDa being the major allergens, recognized by more than 50% of the tested sera. Notably, these proteins were identified as Copt f 7, Copt f 9, and Copt f 3 through inhibition assay using Per a 7, Per a 9, and Per a 3 as inhibitors, respectively. The nucleotide sequencing and homology search revealed high conservation of the major termite allergens Copt f 7, Copt f 9, and Copt f 3 with their respective counterparts in cockroaches, shrimp, and house dust mites. Termite tropomyosin (Copt f 7) showed 99–100% identity with cockroach allergens (Per a 7 and Bla g 7) and 80–83% identity with shrimp and mite allergens. Similarly, termite arginine kinase (Copt f 9) displayed 98% identity with cockroach allergens (Per a 9 and Bla g 9) and 83–91% identity with shrimp and mite allergens. The hemocyanin-like protein (Copt f 3) had lower identities with cockroach allergens (Per a 3 and Bla g 3), at 61% and 57%, respectively. Another possibility is that termite deaths in uninfested homes release alate allergens, potentially exposing a wider community and posing a greater public health concern.

There were limitations in this study. First, the sample size of 16 subjects was relatively small, which may limit the generalizability of our findings. A larger cohort would provide more robust data and potentially reveal additional insights into the prevalence and impact of termite allergies. Second, the study was conducted in specific geographic location (Taichung), which has a subtropical humid climate. Regional differences in termite species distribution and indoor allergen exposure may affect the applicability of our results to other areas. As this study focused on *C. formosanus* and *C. gestroi,* other termite species may also be relevant in other regions, and their allergenic potential was not assessed. Expanding the research to include a broader range of termite species would enhance the understanding of termite allergen diversity. Third, the study did not include longitudinal data to assess the stability of allergic sensitization and symptoms over time. Long-term follow-up studies would be valuable in understanding the chronicity and progression of termite allergies. Fourth, due to limited resources, we did not test the cross-reactivity of Per a 11 to Per a 20 with termites, so the cross-reactions of these allergens with termites could not be determined in this study. Lastly, there was a potential bias in subject selection as they were subjects worked in the termite lab. This may have introduced a selection bias as their exposure levels might have differed from those of the general population. Addressing these limitations in future research would help validate and expand upon our findings, leading to a more thorough understanding of termite allergy and its clinical relevance.

## Conclusions

In conclusion, our study underscores the importance of recognizing termites as a novel indoor allergenic source and the necessity for further research to develop effective diagnostic and therapeutic strategies. Addressing the identified limitations and expanding the scope of future studies will enhance our understanding of termite allergenicity and its impact on allergic diseases.

## Supporting information

S1 Fig Cross-reactivity of IgE assessed by immunoblot inhibition. Immunoblot inhibition was performed using pooled sera from termite-allergic subjects pre-incubated with recombinant cockroach allergens (Per a 7 or Per a 9) at 150 μg/mL at 4 °C overnight. The pre-absorbed sera were then aplied to membranes containing separated termite crude protein extracts, followed by detection with anti-human IgE antibodies.(A) IgE-binding profi le to termite crude extracts without inhibitor. (B) Inhibition of IgE binding after pre-absorption with recombinant Per a 9. (C) Inhibition of IgE binding after pre-absorption with recombinant Per a 7. The data shown are representative of independent experiments.
(TIFF)

S1 Table Sequences of primers used for the PCR-based cloning of *Coptotermes formosanus* allergens. (DOCX)
