# Supplementary figures and images for: Clinical manifestations and cross-reactions between cockroaches and termites and identification of termites’ major allergens
Source: PLoS One. 2026 Mar 23;21(3):e0342319. doi: 10.1371/journal.pone.0342319 (PMC13008072; doi:10.1371/journal.pone.0342319)

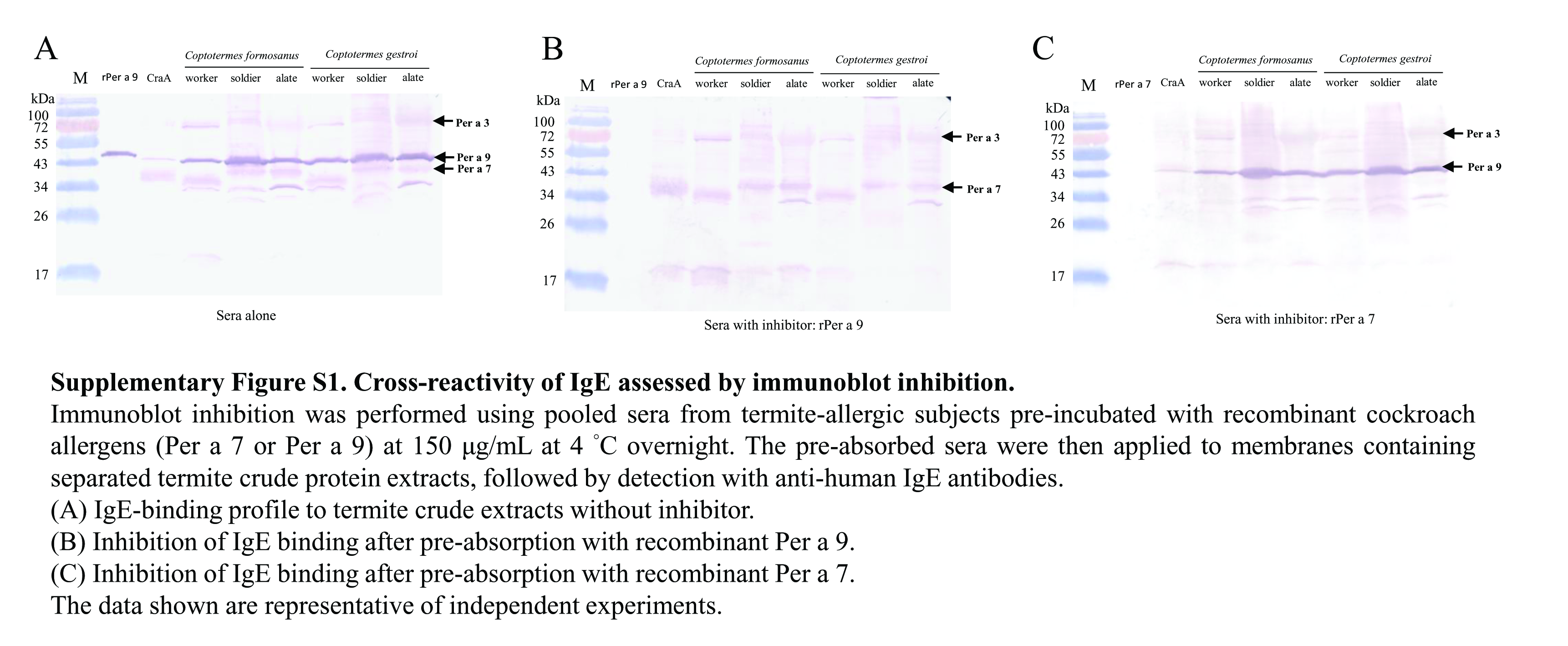

Supplement: S1 Fig — Immunoblot inhibition was performed using pooled sera from termite-allergic subjects pre-incubated with recombinant cockroach allergens (Per a 7 or Per a 9) at 150 μg/mL at 4 °C overnight. The pre-absorbed sera were then aplied to membranes containing separated termite crude protein extracts, followed by detection with anti-human IgE antibodies.(A) IgE-binding profi le to termite crude extracts without inhibitor. (B) Inhibition of IgE binding after pre-absorption with recombinant Per a 9. (C) Inhibition of IgE binding after pre-absorption with recombinant Per a 7. The data shown are representative of independent experiments. (TIFF) [file pone.0342319.s001.tiff]
