# Supplementary material for: Clinical manifestations and cross-reactions between cockroaches and termites and identification of termites’ major allergens
Source: PLoS One. 2026 Mar 23;21(3):e0342319. doi: 10.1371/journal.pone.0342319 (PMC13008072; doi:10.1371/journal.pone.0342319)
Supplement: S1 Table — (DOCX) [file pone.0342319.s002.docx]

| **Supplementary Table S1.** Sequences of primers used for the PCR-based cloning of *Coptotermes formosanus* allergens | | |
| --- | --- | --- |
| Allergen/accession no. | Nucleotide sequences | Theoretical size /pI^b^/Mw^c^ |
| Copt f 3n/ KF718963.1 | F ^1^***GAGCTC***GACCACCACATAGAG | 2007 bp/7.71/79.8 kDa |
|  | R ^2^***AAGCTT***CTAGTGGGTGGTAGT |  |
| Copt f 7n/ KC571878.1 | F ^3^***GGATCC***ATGGATGCGATCAAG | 855 bp/4.56/29.4 kDa |
|  | R ^2^***AAGCTT***TTAGTTGCCAATAAG |  |
| Copt f 9n/^a^ | F ^3^***GGATCC***ATGGTGGATCAAGCA | 1071 bp/5.42/39.8 kDa |
|  | R ^2^***AAGCTT***TTAGAGGGAGCTCTC |  |
| Restriction sites are in italics and priming regions are underlined. ^1^*Sac*I, ^2^*Hind*III, ^3^*Bam*HI. | | |

^a^ Published in Journal of Insect Science, (2020) 20(4): 10; 1–7.

^b^ pI: Isoelectric point

^c^ Mw: Molecular weight
